# Materials of Which the Sun Is Made

**Published:** 1864-03

**Authors:** Wm. Armstrong


MATERIALS OF WHICH THE SUN IS MADE.—Of all the results which science has produced within the last few years, none has been more unexpected than that by which we are enabled to test the materials of which the sun is made, and prove their identity, in part at least, with those of our planet. The spectrum experiments of Bunsen and Kirchhoff have not only shown all this, but they have also corroborated previous conjectures as to the luminous envelope of the sun. I have still to advert to Mr. Nasmyth's remarkable discovery, that the bright surface of the sun is composed of an aggregation of apparently solid forms, shaped like willow leaves or some well known forms of Diatomaceæ, and interlacing one another in every direction. The forms are so regular in size and shape as to have led to a suggestion from one of our profoundest philosophers of their being organisms, possibly even partaking of the nature of life, but, at all events, closely connected with the heating and vivifying influences of the sun. These mysterious objects, which, since Mr. Nasmyth discovered them, have been seen by other observers as well, are computed to be each not less than 1,000 miles in length and about 100 miles in breadth. The enormous chasms in the sun's photosphere, to which we apply the diminutive term "spots," exhibit the extremities of those leaf-like bodies pointing inwards, and fringing the sides of the cavern far down into the abyss. Sometimes they form a sort of rope or bridge across the chasm, and appear to adhere to one another by lateral attraction. I can imagine nothing more deserving the scrutiny of observers than these extraordinary forms. The sympathy also which appears to exist between forces operating in the sun and magnetic forces belonging to the earth merits a continuance of that close attention which it has already received from the British Association, and of labors such as General Sabine has with so much ability and effect devoted to the elucidation of the subject. I may here notice that most remarkable phenomenon which was seen by independent observers at two different places on the first of September, 1859. A sudden outburst of light, far exceeding the brightness of the sun's surface, was seen to take place, and sweep like a drifting cloud over a portion of the solar face. This was attended with magnetic disturbances of unusual intensity, and with exhibitions of aurora of extraordinary brilliancy. The identical instant at which the effusion of light was observed was recorded by an abrupt and strongly marked deflection in the self-registering instruments at Kew. The phenomenon as seen was probably only part of what actually took place, for the magnetic storm in the midst of which it occurred commenced before and continued after the event. If conjecture be allowable in such a case, we may suppose that this remarkable event had some connection with the means by which the sun's heat is renovated. It is a reasonable supposition that the sun was at that time in the act of receiving a more than usual accession of new energy; and the theory which assigns the maintenance of its power to cosmical matter plunging into it with that prodigious velocity which gravitation would impress upon it, as it approached to actual contact with the solar orb, would afford an explanation of this sudden exhibition of intensified light in harmony with the knowledge we have now attained that arrested motion is represented by equivalent heat. Telescopic observations will probably add new facts to guide our judgment on this subject, and, taken in connection with observations on terrestrial magnetism, may enlarge and correct our views respecting the nature of heat, light and electricity. Much as we have yet to learn respecting these agencies, we know sufficient to infer that they cannot be transmitted from the sun to the earth except by communication from particle to particle of intervening matter. Not that I speak of particles in the sense of the atomist. Whatever our views may be of the nature of particles, we must conceive them as centres invested with surrounding forces. We have no evidence, either from our senses or otherwise, of these centres being occupied by solid cores of indivisible incompressible matter essentially distinct from force. Dr. Young has shown that even in so dense a body as water, these nuclei, if they exist at all, must be so small in relation to the intervening spaces, that 100 men distributed at equal distances over the whole surface of England would represent their relative magnitude and distance. What, then, must be these relative dimensions in highly rarefied matter? But why encumber our conceptions of material forces by this unnecessary imagining of a central molecule? If we retain the forces and reject the molecule, we shall still have every property we can recognize in matter by the use of our senses or by the aid of our reason. Viewed in this light, matter is not merely a thing subject to force, but is itself composed and constituted of force.—SIR WM. ARMSTRONG *at Meeting of British Association.*

